# Supplementary material for: Residual insecticide surface treatment for preventing malaria: a systematic review protocol
Source: Syst Rev. 2023 Jun 1;12:89. doi: 10.1186/s13643-023-02259-5 (PMC10233908; doi:10.1186/s13643-023-02259-5)
Supplement: Supplementary file 2 — Additional file 2. Data extraction form. [file 13643_2023_2259_MOESM2_ESM.pdf]

Data Extraction Form

Study characteristics (Study reference)	
**DESIGN**	
Lead Author	
Publication year	
Time period study was conducted	
Design	
Method of Randomization/ matching or other	
Unit of allocation	
Number of units	
Adjustment for clustering	
Outcomes assessed	
**SETTING**	
Country	
Site/s (town/settlements/region)	
Seasonality of transmission	
Level of transmission	
Study site (e.g., rural/ urban/ peri-urban/ level of urbanicity)	
Vector species and vector profile details (i.e., behaviours, resistance profile, parity, sporozoite rates and all other reported information)	
Malaria species	
**PARTICIPANTS (those who received the intervention and on whom impact was measured)**	
Total trial participants in each group/arm (including number of exclusions and reasons)	
Number of clusters per group/arm	
Participants who received the intervention (if provided) and participants on whom the impact was measured	
Recruitment method	
Recruitment rates	
Eligibility criteria	
Characteristics and Demographics (age, sex, ethnicity, SES, time as resident, and all other reported demographics)	
Frequency of travel in the last month	
Cluster details including buffer sizes between	

**clusters, other indication of dilution effects**	
**INTERVENTION and COMPARISON**	
**Insecticide brand, type, formulation (including active ingredient), dose, duration (this includes timing and frequency of application)**	
**Insecticide application method(s) and strategy e.g., spray, paint, treated materials, wallpaper)**	
**Personnel applying the intervention**	
**Target area (inside/outside)**	
**Coverage of household (full, selective/partial)**	
**Coverage across cluster/site/jurisdiction**	
**Length of intervention, including number of rounds of spraying/application per year/ season**	
**Type of dwelling (e.g., fixed or temporary) and construction material of surfaces insecticides applied to (e.g., cement, brick or mud walls, canvas etc)**	
**Time to start intervention after index case**	
**Human behaviour (e.g., sleeping behaviour, re-plastering of houses)**	
**Details of Comparison**	
**How intervention and comparison was measured**	
**Coverage across cluster/site/jurisdiction for the comparison**	
**Background interventions (all reported in primary study – e.g., spraying prior to treatment period and other malaria or vector-specific control interventions [indoor**	

**surface treatment, nets/ other insecticides/ barrier], cointerventions, treatment of individuals that may impact outcome)**
**Costs**
**Resources needed/ used**
**OUTCOME X (repeat as necessary for each outcome)**
**Name / Definition**
**Assessment Metric**
**Events**
**Total (or unit time)**
**Time of outcome assessment**
**Results (i.e., main results – epidemiological and/ or entomological, unadjusted and adjusted, secondary outcomes, subgroups, and clusters)**
**Effect type**
**Unintended benefits**
**Harms**
**ADDITIONAL DATA**
**Other contextual information present/measured/reported) (feasibility, acceptability, preferences/values, impact on equity)**
**Entomological outcomes measured (list)**
**Other**
**Source of funding**
**Possible conflicts of interest**
